# Ruptured solid pseudopapillary neoplasm in children: a 20-year single-center experience

**DOI:** 10.3389/fped.2025.1738046

**Published:** 2026-01-08

**Authors:** Suhyeon Ha, Soo-Min Jeong, Hyunhee Kwon, Jung-Man Namgoong

**Affiliations:** Division of Pediatric Surgery, Asan Medical Center Children's Hospital, Asan Medical Center, University of Ulsan College of Medicine, Songpa-gu, Seoul, Republic of Korea

**Keywords:** pancreatic tumor in children, peritoneal dissemination, recurrence, solid pseudopapillary neoplasm, tumor rupture

## Abstract

**Purpose:**

Solid pseudopapillary neoplasm (SPN) is a rare pancreatic tumor with an excellent prognosis after resection. Tumor rupture is uncommon and may increase the risk of peritoneal spread, but data in children are extremely limited. This study evaluated clinicopathologic features, operative outcomes, and recurrence patterns in ruptured SPN in children over 20 years.

**Methods:**

We retrospectively reviewed seven children (<18 years) with histologically confirmed ruptured SPN who underwent resection at a tertiary center between 2004 and 2024. Clinicopathologic, operative, and follow-up data were analyzed. Recurrence was categorized as suspected (radiologic) or confirmed (histologic).

**Results:**

All patients were female (median age 12 years). Median tumor size was 7.4 cm, mostly arising from the pancreatic body or tail. Rupture was spontaneous in 42.9% and trauma-related in 57.1%; no iatrogenic rupture events occurred. Distal pancreatectomy was performed in 71.4%. Suspected recurrence occurred in five patients (71.4%), during a median follow-up of 47 months; however, confirmed recurrence was identified in two (28.6%), both with peritoneal dissemination. Both confirmed recurrence cases involved tumors larger than 10 cm with peripancreatic soft tissue extension, although no statistical associations can be inferred due to the small sample size.

**Conclusion:**

Ruptured SPN in children carries a measurable risk of true peritoneal recurrence, but radiologic findings may overestimate relapse. Tumor size and peripancreatic extension may help stratify recurrence risk. Complete resection, thorough peritoneal assessment, and long-term surveillance are essential. Multicenter studies are needed to refine management strategies.

## 1 Introduction

Solid pseudopapillary neoplasm (SPN) of the pancreas is a rare, low-grade malignant tumor accounting for approximately 1%–3% of all pancreatic neoplasms (1–3). It predominantly affects young females (4) and generally carries a favorable prognosis after complete surgical resection (5).

Tumor rupture in SPN is uncommon, and reports involving children with SPN are exceedingly rare. Rupture is clinically significant because it may increase the risk of peritoneal dissemination and subsequent tumor recurrence (6, 7). Although several case reports and small case series have described ruptured SPN, data specific to the pediatric population remain scarce, particularly concerning long-term oncologic outcomes (8). Due to the rarity of this condition, evidence guiding optimal management and postoperative surveillance in children is limited. In particular, recurrence risk, histopathologic correlations, and surgical outcomes have not been comprehensively evaluated in pediatric cases of ruptured SPN.

This study presents a 20-year, single-center experience with children diagnosed with ruptured SPN, focusing on clinicopathologic features, operative management, and long-term outcomes. We further describe recurrence patterns and potential prognostic factors to inform surgical decision-making and refine surveillance strategies for this rare pediatric entity.

## 2 Materials and methods

### 2.1 Study design and patient selection

This retrospective study included children (≤18 years) with ruptured SPN who underwent surgical resection at Asan Medical Center between January 2004 and December 2024. Inclusion criteria were: (1) histopathologic confirmation of SPN and (2) radiologic or intraoperative evidence of tumor rupture. Tumor rupture was defined using both radiologic and intraoperative criteria.

Radiologic rupture was diagnosed when high-attenuation intraperitoneal fluid, hemoperitoneum, capsular discontinuity, or peritumoral fluid–fluid levels were present on computed tomography (CT), or magnetic resonance imaging (MRI). Intraoperative rupture was defined as visible capsular disruption, adherent clot, intraperitoneal tumor debris, or intraoperative spillage.

Rupture mechanisms were categorized as traumatic (blunt trauma or sports-related injury) or iatrogenic, the latter including biopsy-related disruption or intraoperative capsular violation. Diagnostic-procedure–related events were classified as iatrogenic rupture.

**Figure 1:**
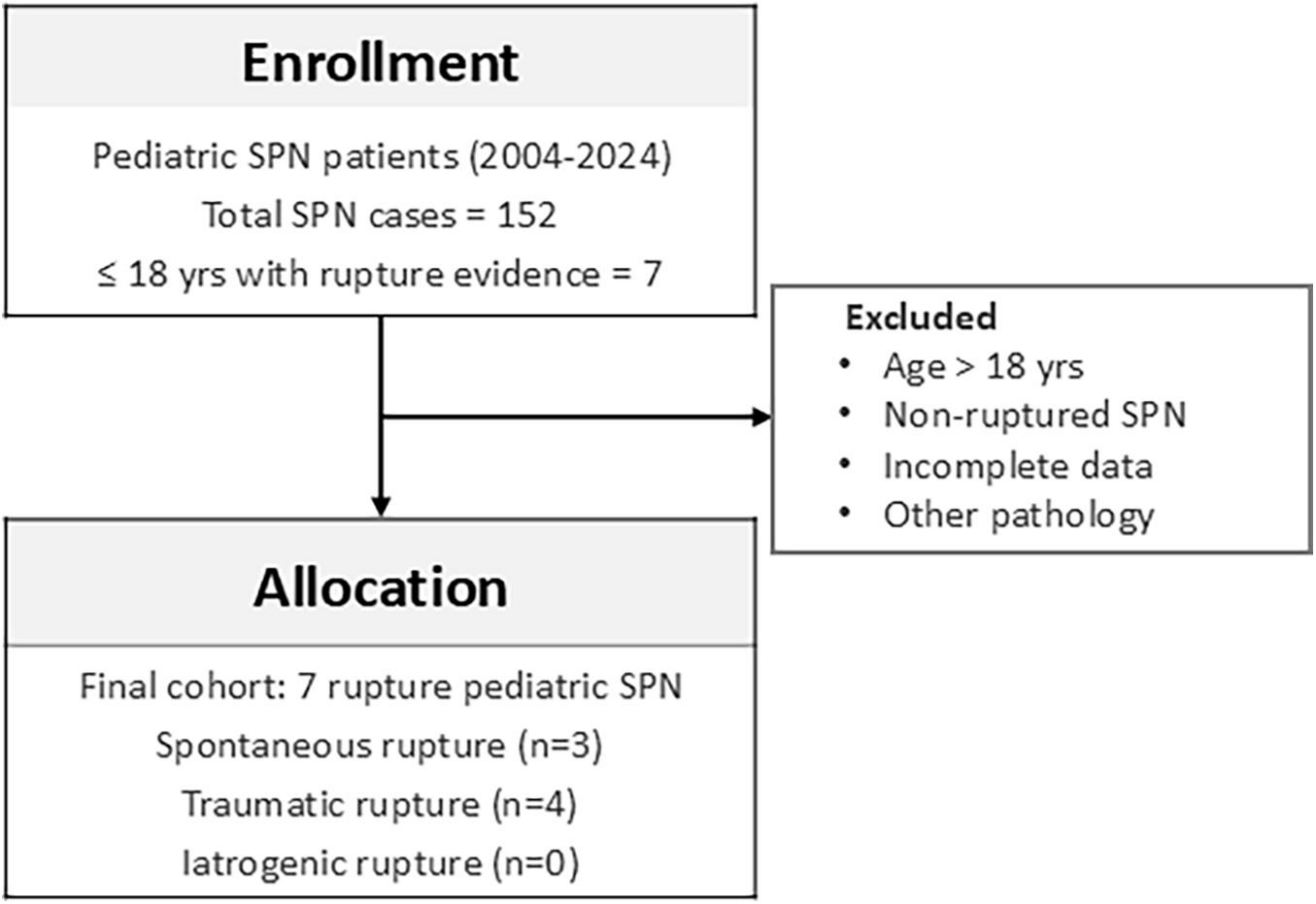
Flow diagram of patient selection. Among 152 patients with solid pseudopapillary neoplasm (SPN) treated between 2004 and 2024, seven children (≤18 years) with radiologic or intraoperative evidence of rupture were included.

Patients were excluded if they had non-ruptured SPN, lacked histologic confirmation, or had incomplete clinical, pathological, or follow-up data. A flow diagram summarizing patient selection is presented in Figure 1.

This study was approved by the Institutional Review Board of Asan Medical Center (IRB No. 2025-1306) and conducted in accordance with the principles of the Declaration of Helsinki. The requirement for informed consent was waived owing to the retrospective nature of the study.

### 2.2 Data collection

Demographic variables included sex, age, admission and discharge dates, and follow-up duration. Clinical data comprised presenting symptoms and their duration. Tumor characteristics, including size, location, and rupture mechanism, were recorded along with details of preoperative imaging and laboratory evaluation. No patient underwent preoperative biopsy, as biopsy is not routinely performed in SPN in children at our institution. Operative variables included the type of surgical procedure, operative time, concomitant organ resection, intraoperative transfusion, and length of hospital stay.

### 2.3 Operative approach and surgical technique

Laparoscopic resection was considered for patients with smaller, well-circumscribed tumors without extensive preoperative evidence of rupture, whereas open surgery was preferred when rupture was strongly suspected, tumor size was large, or adjacent organ involvement or dense adhesions were anticipated. Surgeon experience and intraoperative judgment also contributed to approach selection. Standard operative principles included complete peritoneal exploration, careful handling of the tumor to avoid spillage, extensive lavage when rupture was present or suspected, and confirmation of negative margins.

### 2.4 Pathologic evaluation

Pathologic assessment included tumor size, location, encapsulation, necrosis, hemorrhage, and mitotic activity. Margin status was reviewed separately for pancreatic transection and peripancreatic radial margins. The presence of peripancreatic soft tissue invasion, lymphovascular invasion, perineural invasion, lymph node metastasis, and peritoneal seeding at the time of surgery was also evaluated.

Immunohistochemical (IHC) staining was routinely performed for diagnostic confirmation. The IHC panel included β-catenin (nuclear positivity), CD10, vimentin, and E-cadherin, as well as neuroendocrine markers such as synaptophysin.

### 2.5 Postoperative outcomes and follow-up

Postoperative complications were classified according to the Clavien–Dindo grading system (9). Pancreatic fistula and fluid collection were defined in accordance with the criteria established by the International Study Group of Pancreatic Surgery (ISGPS) (10). Recurrence was categorized as suspected (radiologic lesion concerning for SPN) or confirmed (histologically proven viable SPN). For suspected or confirmed recurrence, recurrence site, management, and survival status were documented. Surveillance consisted of ultrasound, CT, or MRI every 6–12 months. Follow-up duration was calculated from the date of surgery to the last visit or death.

### 2.6 Statistical analysis

Only descriptive statistics were used because of the small sample size and limited number of recurrence events. Continuous variables are presented as medians with interquartile range (IQR), and categorical variables are reported as counts and percentages. No inferential statistical tests were performed.

## 3 Results

### 3.1 Patient characteristics

During the study period, our institution treated 152 children with SPN, among whom seven patients (4.6%) presented with tumor rupture and were included in this series (Table 1). All patients were female. The median age at the time of surgery was 12 years (IQR 10.5–13.5), and the median body mass index was 17.9 (IQR 17.0–20.5). Abdominal pain was universal, while nausea/vomiting and fever occurred in 57.1% and 42.9%, respectively. Symptoms developed over a median duration of 1.0 month (IQR 0.2–1.5).

**Table 1:** Clinical characteristics of children with ruptured solid pseudopapillary neoplasm.

Variable	Findings (*N* = 7)
Sex	
Male	0
Female	7 (100%)
Age, years, median [IQR]	12 [10.5–13.5]
BMI, median [IQR]	17.9 [17.0–20.5]
Preoperative symptom	
Abdominal pain	7 (100%)
Nausea, vomiting	4 (57.1%)
Fever	3 (42.9%)
Symptom length, month, median [IQR]	1 [0.2–1.5]
Rupture mechanism	
Spontaneous	3 (42.9%)
Traumatic	4 (57.1%)
Iatrogenic	0
Tumor size, cm, median [IQR]	7.4 [5.4–8.4]
Tumor location	
Head	1 (14.3%)
Body	3 (42.9%)
Tail	3 (42.9%)
Vessel invasion	
Splenic vessels	1 (14.3%)
Synchronous seeding	1 (14.3%)
Synchronous metastasis	1 (14.3%)
Intratumoral hemorrhage	7 (100%)
Pseudocyst	0
Preoperative pancreatitis	3 (42.9%)

SPN, solid pseudopapillary neoplasm; BMI, body mass index; IQR, interquartile range.

Median tumor diameter was 7.4 cm (IQR, 5.4–8.4), most commonly arising from the pancreatic body or tail (each 42.9%). Rupture was spontaneous in 42.9% and trauma-related in 57.1%; no iatrogenic rupture events occurred in this cohort. Preoperative pancreatitis was observed in three patients (42.9%). At presentation, one patient (14.3%) had synchronous liver and para-aortic lymph node metastasis, and another (14.3%) had evidence of intra-abdominal seeding. Intratumoral hemorrhage was identified in all cases. Both metastatic lesions showed classic SPN morphology without high-grade atypia, increased mitotic activity, or necrosis.

### 3.2 Operative details and postoperative outcomes

Distal pancreatectomy (± splenectomy) was performed in 71.4% of patients, with one enucleation and one pylorus-preserving pancreaticoduodenectomy (Table 2). Three procedures were open, three laparoscopic, and one robotic. Concomitant organ resection occurred in 42.9%. Median operative time was 262 min (IQR, 191–361) without intraoperative transfusion. Three procedures were open, three were laparoscopic, and one was robotic. Peritoneal seeding was identified intraoperatively in 42.9%, occurring in both open and laparoscopic cases. Rates of suspected and confirmed recurrence were similar between laparoscopic and open approaches, and no iatrogenic rupture or tumor spillage occurred during minimally invasive surgery. All postoperative complications were minor (Clavien–Dindo I–II), and pancreatic fistula occurred in 57.1% (biochemical 28.6%, grade B 28.6%). No grade C fistula or major morbidity was observed. Oral intake resumed at a median of 7 days, and median hospitalization was 21 days (IQR, 15.5–28.0).

**Table 2:** Operative findings and postoperative outcomes in children with ruptured SPN.

Variable	Findings (*N* = 7)
Operative approach	
Open^a^	3 (42.9%)
Laparoscopy	3 (42.9%)
Robot	1 (14.3%)
Procedure type	
Distal pancreatectomy ± splenectomy	5 (71.4%)
Enucleation	1 (14.3%)
PPPD	1 (14.3%)
Pancreas resection method	
Endoscopic stapler	2 (28.6%)
Energy device	2 (28.6%)
Others	3 (42.9%)
Operative time, min, median [IQR]	262 [191–361]
Intraoperative transfusion	0
Spleen management	
Total splenectomy	1 (14.3%)
Warshaw	1 (14.3%)
Intraoperative seeding	3 (42.9%)
Omentum	2 (28.6%)
Mesentery	1 (14.3%)
Concomitant organ resection	3 (42.9%)
Complications	4 (57.1%)
C–D minor (C–D I-II)	4 (57.1%)
C–D major (C–D III-IV)	0
Pancreatic leak	
None	3 (42.9%)
Biochemical leak	2 (28.6%)
Grade B	2 (28.6%)
Grade C	0
Post-pancreatectomy hemorrhage	0
Fluid collection	3 (42.9%)
Abscess	1 (14.3%)
Ileus	2 (28.6%)
Postoperative diet start, days, median [IQR]	7 [7–10]
Length of stay, days, median [IQR]	21 [15.5–28]

a One laparoscopic case was converted to open surgery due to intraoperative adhesion.

SPN, solid pseudopapillary neoplasm; PPPD, pylorus-preserving pancreaticoduodenectomy; C–D, Clavien–Dindo classification; IQR, interquartile range.

No patient developed new-onset diabetes or required insulin therapy. HbA1c was assessed in two patients during follow-up, with values of 5.7% and 5.5%. No patient experienced exocrine insufficiency, required pancreatic enzyme supplementation, or demonstrated growth impairment.

### 3.3 Pathologic findings

Tumor size ranged from 4.2 to 10.6 cm (Table 3). Most tumors were encapsulated, although capsular disruption or overt rupture occurred in two specimens. Hemorrhage and necrosis were frequent. Peripancreatic soft tissue invasion was seen in 57.1%. Margins were negative in all except one radial margin. No lymphovascular or perineural invasion was detected, and all lymph nodes examined were negative. One patient had synchronous omental metastasis.

**Table 3:** Histopathologic features of resected tumors in children with ruptured SPN.

Case	Size (cm)	Location	Encapsulation	Necrosis/Hemorrhage	Vessel invasion	LVI	PNI	Soft tissue extension	Margin status	LN metastasis	Peritoneal seeding	IHC profile
1	5.4	Tail	Present, focally disrupted	Focal hemorrhage	Splenic a,v. abutting	No	No	Yes (peripancreatic)	Peripancreatic margin focally involved; pancreatic margin free	0/1	No	*β*-catenin (+), E-cadherin (–), Synaptophysin (focal +)
2	6.5	Tail	Present	Multifocal hemorrhage, necrosis	Splenic a,v. abutting	No	No	No	Clear resection margin	0/4	No	β-catenin (+), E-cadherin (–)
3	10	Body	Disrupted	Multifocal hemorrhage, necrosis	Splenic v invasion	No	No	Yes (peripancreatic)	Clear resection margin	0/2	Yes	β-catenin (+), E-cadherin (–), Synaptophysin (–)
4	8.5	Body	Present, ruptured	Spotty necrosis, extensive hemorrhage	PV abutting	No	No	Unavailable	Clear resection margin	0	Omental metastasis (1.7 cm)	β-catenin (+), E-cadherin (–), Synaptophysin (focal +), Chromogranin (–)
5	4.2	Head	Present	Hemorrhage, necrosis	None	No	No	Yes (peripancreatic)	Clear resection margin	0/13	No	β-catenin (+), E-cadherin (–)
6	4.2	Body	Present	Hemorrhage	None	No	No	No	Clear resection margin	Not described	No	Not described
7	10.6	Tail	Present	Multifocal hemorrhage, necrosis	None	No	No	Yes (peripancreatic)	Clear resection margin	0/5	No	β-catenin (+), E-cadherin (–), Synaptophysin (focal +), Chromogranin (–)

SPN, solid pseudopapillary neoplasm; LVI, lymphovascular invasion; PNI, perineural invasion; LN, lymph node; PV, portal vein; IHC, immunohistochemistry; a, artery; v, vein.

Ki-67 proliferation index was not available for any patient.

Immunohistochemical (IHC) staining consistently demonstrated nuclear β-catenin positivity and E-cadherin loss; synaptophysin expression was variable and chromogranin was negative. Ki-67 index was not available for any patient, as Ki-67 staining is not routinely performed at our institution for children with SPN.

### 3.4 Recurrence and long-term outcomes

For context, among children with non-ruptured SPN treated during the same period, **7** patients also experienced recurrence during long-term follow-up. Although these non-ruptured cases were not included in the current analysis, their existence demonstrates that recurrence is not exclusive to ruptured SPN.

**Figure 2:**
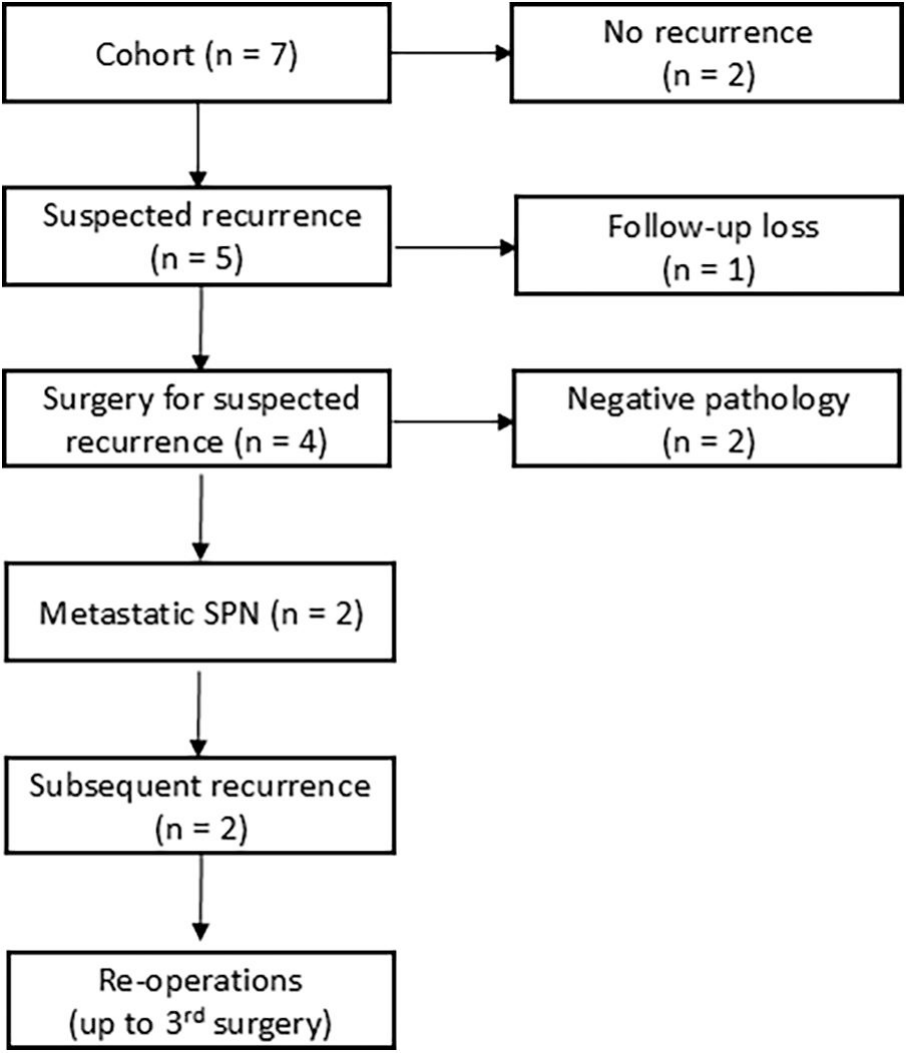
Flow diagram summarizing suspected and confirmed recurrence events and their management in children with ruptured SPN. Five patients developed suspected recurrence; four underwent reoperation, with confirmed recurrence identified in two. One patient with suspected recurrence was lost to follow-up.

Over a median 47-month follow-up (range, 23–60), suspected recurrence arose in five patients (71.4%) (Table 4). One of these patients was lost to follow-up. The remaining four underwent reoperation: confirmed recurrence was identified in two patients, both showing peritoneal dissemination, whereas the other two had no viable tumor and were diagnosed with reactive fibroinflammatory changes (Figure 2). These two non-confirmed cases were subsequently followed with serial CT or MRI and clinical assessment, during which the suspected lesions remained radiologically stable or regressed. Both patients with confirmed recurrence experienced multiple relapses requiring repeated surgical resections.

**Table 4:** Patterns of suspected and confirmed recurrence during long-term follow-up in children with ruptured SPN.

Case	Suspected recurrence (month)	Time to Recurrence	Operation site(s)	Reoperation	Confirmed recurrence	No. of reoperations	Death	Last follow-up (month)
1	Yes	13	Pancreatic resection margin, LN #4	Yes	Low-grade spindle cell proliferative lesion	1	Alive	47
2	Yes	11	Abdominal wall, LN #4	Yes	Stromal fibrosis with foreign body reaction	1	Alive	25
3	Yes	1	Liver, para-aortic lymph node	Yes	**Metastatic SPN**	3	Alive	23
4	Yes	53	Peritoneum	No^a^		0	Alive	53
5	No	–	–	No		0	Alive	55
6	No	–	–	No		0	Alive	60
7	Yes	38	Peritoneum, trocar site, omentum, cul-de-sac, uterus, pelvic cavity	Yes	**Metastatic SPN**	2	Alive	47

a Lost to follow-up.

SPN, solid pseudopapillary neoplasm; LN, lymph node.

**Figure 3:**
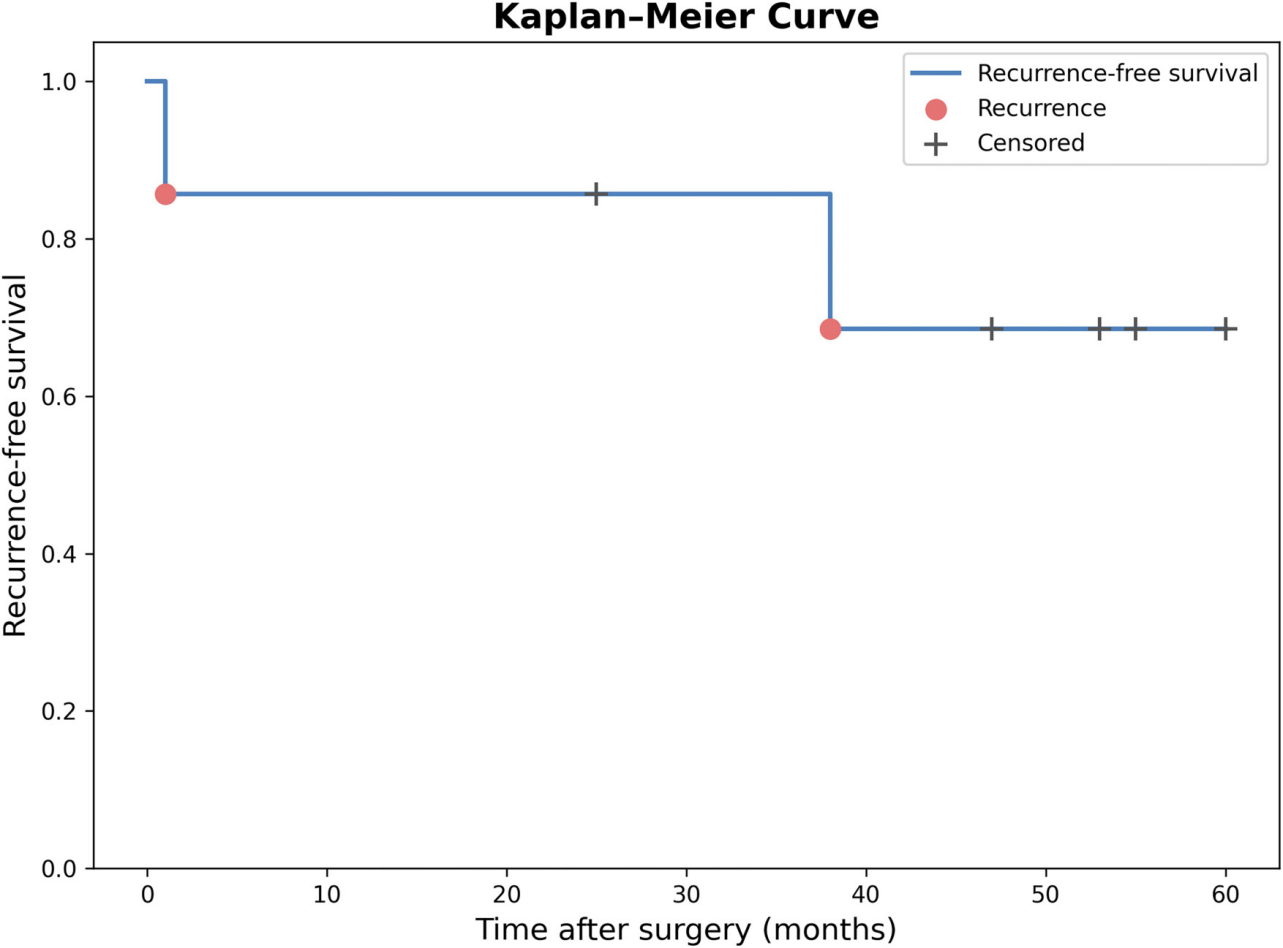
Kaplan–meier curve for recurrence-free survival in children with ruptured SPN. Confirmed recurrence was identified in two patients. Censored observations indicate patients without recurrence at final follow-up.

**Figure 4:**
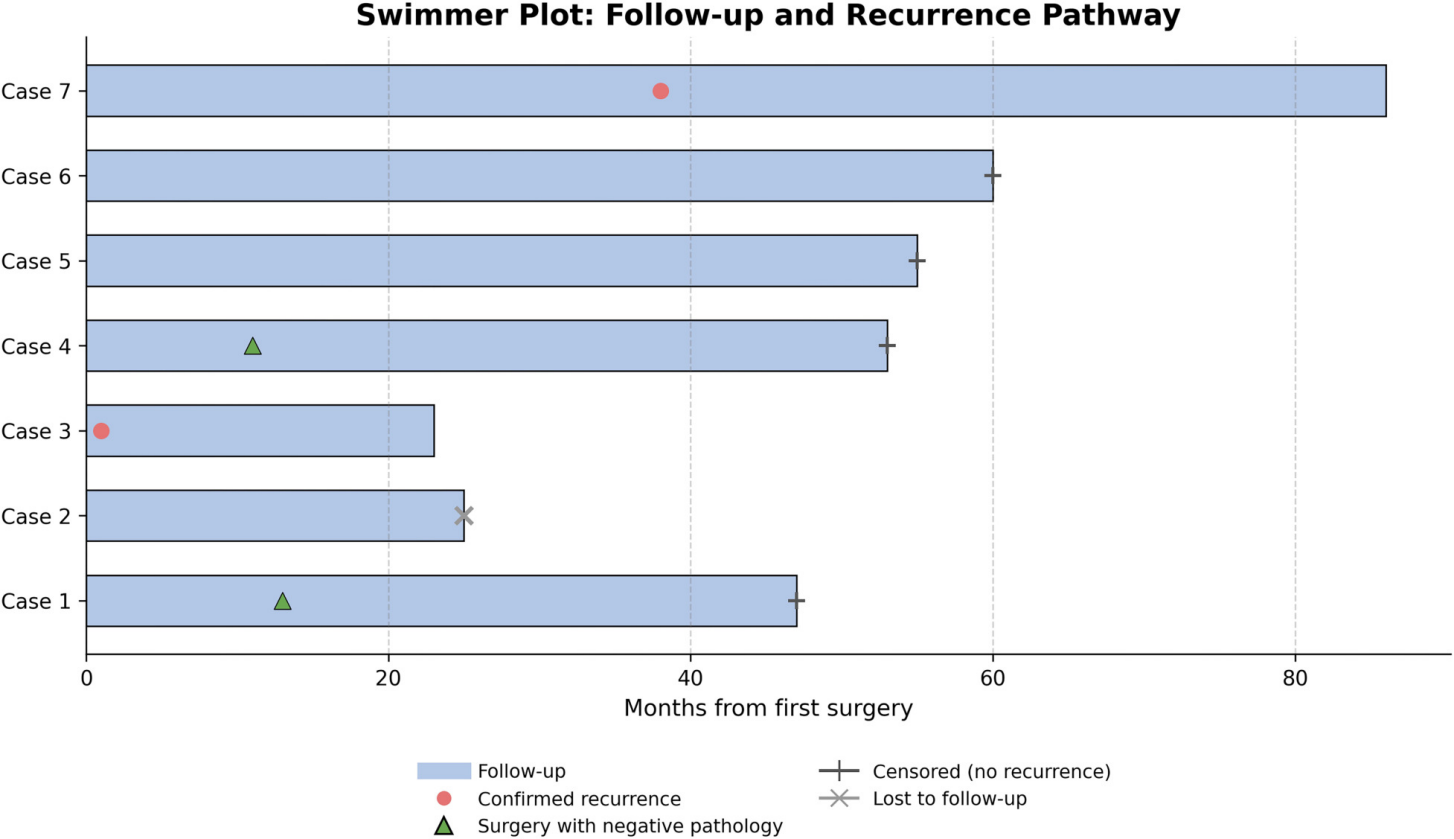
Timeline of follow-up, suspected recurrence, and confirmed recurrence events in children with ruptured SPN. Red circles indicate confirmed recurrence, green triangles indicate reoperation without viable tumor, cross marks represent loss to follow-up, and short vertical bars indicate censoring at last follow-up.

No patient received adjuvant therapy, including chemotherapy or radiotherapy, either at the time of initial surgery or at the time of recurrence. All patients were alive at last follow-up. Kaplan–Meier analysis of recurrence-free survival is shown in Figure 3. Figure 4 illustrates individual patient follow-up courses, highlighting the timing of suspected and confirmed recurrence and reoperations.

A descriptive comparison of clinicopathologic and operative characteristics between patients with confirmed recurrence (*n* = 2) and those without recurrence (*n* = 5) is summarized in Table 5.

**Table 5:** Descriptive comparison of clinicopathologic and operative features between children with confirmed recurrence and those without recurrence after ruptured SPN resection.

Variable	Recurrence	Recurrence	Non-recur	Non-recur	Non-recur	Non-recur	Non-recur
	Case 3	Case 7	Case 1	Case 2	Case 4	Case 5	Case 6
Tumor size (cm)	10	10.6	5.4	6.5	8.5	4.2	4.2
Tumor location	Body	Tail	Tail	Tail	Body	Head	Body
Rupture mechanism	Spontaneous	Traumatic	Traumatic	Spontaneous	Spontaneous	Spontaneous	Traumatic
Soft tissue extension	Yes (peripancreatic)	Yes (peripancreatic)	Yes (peripancreatic)	No	Not available	Yes (peripancreatic)	No
Margin status	R0	R0	R1 (peripancreatic)^a^, R0 (pancreatic)	R0	R0	R0	R0
Intraoperative seeding	Yes (omentum)	No	No	No	Yes (omentum)	Yes (mesentery)	No
Adjuvant therapy	None	None	None	None	None	None	None

a R1 margin status indicates focal involvement of the peripancreatic radial margin.

Values are descriptive only; no statistical comparisons were performed due to small sample size.

### 3.6 Risk factor analysis

As summarized in Table 5, both patients with confirmed recurrence had tumors >10 cm and peripancreatic soft tissue extension, whereas these features were less frequent in non-recurrent cases; however, given the small sample size, no statistical associations can be inferred. Other histologic variables—including capsule integrity, lymphovascular or perineural invasion, and nodal status—did not correlate with recurrence. Notably, one patient recurred despite lacking conventional aggressive features, suggesting that rupture itself and tumor burden may contribute to relapse risk**.**

## 4 Discussion

To our knowledge, this study represents the largest case series to date focusing exclusively on children with ruptured SPN—an exceedingly rare clinical entity. With a median follow-up of 47 months, we conducted a comprehensive assessment encompassing clinical presentation, operative findings, histopathologic characteristics, and long-term outcomes. Suspected recurrence arose in five patients, whereas confirmed recurrence was identified in only two (28.6%). Among the cases with peritoneal seeding, two were attributable to tumor rupture at presentation, whereas the remaining events represented later disease progression. Both confirmed cases involved large tumors exceeding 10 cm and exhibited peripancreatic soft tissue extension, whereas other histologic variables—such as capsule integrity, lymphovascular invasion, and lymph node involvement—were not consistently associated with relapse. Notably, our clear distinction between suspected and confirmed recurrence offers a novel perspective and underscores the importance of cautious interpretation of postoperative surveillance imaging.

Although SPN generally carries an excellent prognosis, recurrence or metastasis may occur in a small subset of patients (11). Pooled analyses of long-term data from non-ruptured SPN cohorts have reported recurrence rates between 10% and 15% (12, 13). A comprehensive meta-analysis identified several adverse prognostic factors, including tumor diameter greater than 5 cm, lymphovascular or nodal invasion, synchronous metastasis, and positive resection margins. Subsequent studies further proposed that tumors exceeding 8 cm, the presence of microscopic malignant features, synchronous metastases, and an elevated Ki-67 index (>4%) were predictive of recurrence (14). More recently, a large retrospective Chinese series involving 195 patients found incomplete tumor encapsulation to be an independent predictor of “malignant SPN” behavior, although the overall 10-year disease-free survival after R0 resection remained favorable at approximately 94% (15). Consistent with these observations, multiple systematic reviews and contemporary cohort studies have emphasized that, despite its classification as a low-grade neoplasm, a subset of SPNs display distinctly aggressive biological behavior (16, 17). Our study differs from these prior investigations by focusing specifically on pediatric cases of ruptured SPN and by explicitly differentiating between radiologic suspicion and pathologically confirmed recurrence. Unlike earlier reports that often relied solely on imaging or clinical impression, our findings demonstrate that not all radiologic abnormalities represent confirmed recurrence. This distinction carries substantial clinical importance when determining surveillance intensity and the need for surgical re-exploration.

Our findings, in conjunction with previous reports, provide several clinically meaningful insights. Rigorous long-term surveillance remains essential in the management of ruptured SPN. Although pathologically confirmed recurrence occurred in only a small subset of patients, radiologic suspicion arose in the majority (five of seven), highlighting the need for careful longitudinal assessment that integrates clinical evaluation, imaging interpretation, and—when indicated—surgical exploration. A risk-adapted surveillance approach may therefore be warranted. In our cohort, the two confirmed recurrence cases shared tumors >10 cm with peripancreatic soft tissue extension, although the small sample size precludes any statistical inference. Additional prognostic variables identified in broader SPN literature—including tumor size greater than 5–8 cm, positive resection margins, lymphovascular invasion, nodal metastasis, microscopic malignant features, and elevated Ki-67 index—should also inform the intensity and duration of postoperative follow-up in both ruptured and non-ruptured SPN cases (18). In our cohort, Ki-67 was not available because it is not routinely performed for SPN in children at our institution.

Meticulous surgical management is likewise critical in rupture cases. Comprehensive intraoperative exploration of the peritoneal cavity, extensive lavage, and confirmation of negative resection margins may help reduce the risk of peritoneal dissemination. In this context, the choice between laparoscopic and open surgery also warrants consideration. Minimally invasive resection was selectively attempted in patients with smaller, well-circumscribed tumors without extensive preoperative evidence of rupture, whereas open surgery was favored when rupture was strongly suspected, tumor size was large, or adhesions or adjacent organ involvement were anticipated. Importantly, peritoneal seeding was observed in both open and laparoscopic procedures, and rates of suspected or confirmed recurrence were comparable between approaches. No case of iatrogenic rupture or tumor spillage occurred during minimally invasive surgery, supporting its feasibility in carefully selected pediatric patients.

Although two patients presented with synchronous metastases involving the omentum, liver, or para-aortic lymph nodes, all metastatic deposits retained classic SPN morphology without high-grade atypia, increased mitotic activity, or extensive necrosis. Therefore, none fulfilled the WHO 2022 histologic criteria for solid pseudopapillary carcinoma. Confirmed recurrence later developed in two patients, both showing regional or peritoneal dissemination rather than new distant organ metastasis. This observation suggests that even histologically benign-appearing SPNs possess the potential for locoregional relapse, reinforcing the importance of meticulous intraoperative inspection of the entire peritoneal cavity and vigilant postoperative surveillance, including careful assessment of the pelvic cavity. Postoperative pancreatic function was well preserved in all patients, with no cases of endocrine or exocrine insufficiency and normal HbA1c values in the two patients assessed. These findings reinforce that long-term functional outcomes are excellent in pediatric SPN, even in ruptured cases. Notably, hepatic recurrence was among the confirmed relapse sites in our cohort. Because small or subcapsular hepatic lesions may be difficult to detect on CT, particularly in young patients, supplemental MRI should be considered when CT findings are inconclusive or when clinical or biochemical suspicion persists. Implementation of a structured surveillance protocol alternating CT and MRI examinations may enhance postoperative monitoring sensitivity and facilitate earlier detection of subtle or atypical recurrences.

This study has several limitations that warrant consideration. First, the small sample size (*N* = 7) inevitably limits statistical power and precludes robust multivariate analysis. Second, its retrospective, single-center nature may introduce referral bias, while variations in imaging surveillance protocols could have influenced recurrence detection rates. Third, histopathologic data were derived from archival pathology reports rather than standardized re-review of all specimens, potentially introducing reporting heterogeneity. Fourth, one patient was lost to follow-up before possible reoperation, resulting in uncertainty regarding recurrence status.

To strengthen and validate these findings, multicenter collaboration and registry-based studies are needed, particularly in children, in whom ruptured SPN remains rare. Prospective protocols that standardize imaging intervals, surgical decision-making, and criteria for intervention based on risk stratification will be essential for refining postoperative surveillance strategies. In addition, future research integrating molecular and immunohistochemical profiling—including *CTNNB1* mutation status, Ki-67 proliferation index, and hormonal receptor expression—may help elucidate the biological mechanisms underlying recurrence and enable more precise, individualized risk prediction in children with ruptured SPN.

These observations support a risk-adapted surveillance strategy, with prolonged and meticulous imaging follow-up particularly in patients with large tumors or peripancreatic extension.

## 5 Conclusion

In summary, although suspected recurrence was common among children with ruptured SPN, pathologically confirmed recurrence occurred in only a small subset. In our cohort, both confirmed recurrence cases involved tumors larger than 10 cm with peripancreatic soft tissue extension, although these observations are descriptive and limited by the small sample size. These findings highlight the importance of cautious interpretation of postoperative imaging, the value of risk-adapted long-term surveillance, and the necessity of histopathologic confirmation to distinguish true recurrence from radiologic abnormalities. Larger, multicenter studies are needed to better define recurrence risk and guide evidence-based management strategies for this rare clinical entity.

## Data Availability

The original contributions presented in the study are included in the article/Supplementary Material, further inquiries can be directed to the corresponding author.
